# The quaternary structure of human tyrosine hydroxylase: effects of dystonia‐associated missense variants on oligomeric state and enzyme activity

**DOI:** 10.1111/jnc.14624

**Published:** 2018-12-09

**Authors:** Peter D. Szigetvari, Gopinath Muruganandam, Juha P. Kallio, Erik I. Hallin, Agnete Fossbakk, Remy Loris, Inari Kursula, Lisbeth B. Møller, Per M. Knappskog, Petri Kursula, Jan Haavik

**Affiliations:** ^1^ Department of Biomedicine University of Bergen Bergen Norway; ^2^ K.G. Jebsen Centre for Research on Neuropsychiatric Disorders University of Bergen Bergen Norway; ^3^ VIB‐VUB Center for Structural Biology, Vlaams Instituut voor Biotechnologie Brussels Belgium; ^4^ Structural Biology Brussels Department of Bioengineering Sciences Vrije Universiteit Brussel Brussels Belgium; ^5^ Faculty of Biochemistry and Molecular Medicine University of Oulu Oulu Finland; ^6^ Biocenter Oulu University of Oulu Oulu Finland; ^7^ Department of Clinical Science University of Bergen Bergen Norway; ^8^ Applied Human Molecular Genetics Kennedy Center Department of Clinical Genetics Copenhagen University Hospital Rigshospitalet, Glostrup Denmark; ^9^ Center for Medical Genetics and Molecular Medicine Haukeland University Hospital Bergen Norway; ^10^ Division of Psychiatry Haukeland University Hospital Bergen Norway

**Keywords:** enzymatic activity, missense variants, oligomeric structure, SEC‐SAXS, TEM, tyrosine hydroxylase deficiency

## Abstract

**Abstract:**

Tyrosine hydroxylase (TH) is a multi‐domain, homo‐oligomeric enzyme that catalyses the rate‐limiting step of catecholamine neurotransmitter biosynthesis. Missense variants of human TH are associated with a recessive neurometabolic disease with low levels of brain dopamine and noradrenaline, resulting in a variable clinical picture, from progressive brain encephalopathy to adolescent onset DOPA‐responsive dystonia (DRD). We expressed isoform 1 of human TH (hTH1) and its dystonia‐associated missense variants in *E. coli,* analysed their quaternary structure and thermal stability using size‐exclusion chromatography, circular dichroism, multi‐angle light scattering, transmission electron microscopy, small‐angle X‐ray scattering and assayed hydroxylase activity. Wild‐type (WT) hTH1 was a mixture of enzymatically stable tetramers (85.6%) and octamers (14.4%), with little interconversion between these species. We also observed small amounts of higher order assemblies of long chains of enzyme by transmission electron microscopy. To investigate the role of molecular assemblies in the pathogenesis of DRD, we compared the structure of WT hTH1 with the DRD‐associated variants R410P and D467G that are found in vicinity of the predicted subunit interfaces. In contrast to WT hTH1, R410P and D467G were mixtures of tetrameric and dimeric species. Inspection of the available structures revealed that Arg‐410 and Asp‐467 are important for maintaining the stability and oligomeric structure of TH. Disruption of the normal quaternary enzyme structure by missense variants is a new molecular mechanism that may explain the loss of TH enzymatic activity in DRD. Unstable missense variants could be targets for pharmacological intervention in DRD, aimed to re‐establish the normal oligomeric state of TH.

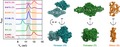

**Abbreviations used**
BH_4_: tetrahydrobiopterin
CA: catecholamine
DA: dopamine
DRD: L‐DOPA‐responsive dystonia
L‐DOPA: L‐dihydroxyphenylalanine
MALS: multi‐angle static light scattering
SAXS: small‐angle X‐ray scattering
SDS‐PAGE: sodium dodecyl sulphate‐polyacrylamide gel electrophoresis
SEC: size‐exclusion chromatography
TEM: transmission electron microscopy
THD: tyrosine hydroxylase deficiency
TH: tyrosine hydroxylase
V_max_: maximal velocity
WT: wild‐type

Tyrosine hydroxylase (TH; EC 1.14.16.2; tyrosine 3‐monooxygenase) is a non‐haeme iron‐ (Haavik *et al*. 1991) and tetrahydrobiopterin (BH_4_)–dependent enzyme that catalyses the rate‐limiting step in catecholamine biosynthesis (Nagatsu *et al*. 1964) through the insertion of a single atom from molecular oxygen onto the amino acid substrate aromatic ring. The remaining oxygen atom is reduced to water in a reaction, in which BH_4_ acts as an electron donor (Roberts and Fitzpatrick 2013).

Along with phenylalanine hydroxylase (PAH) and tryptophan hydroxylases 1 and 2 (TPH1 and TPH2), TH belongs to the protein family of aromatic amino acid hydroxylases (AAAHs) (Grenett *et al*. 1987). Similar to PAH, TPH1 and TPH2, TH in solution is an oligomer of identical subunits, where each subunit contains an N‐terminal regulatory domain, followed by a catalytic core and a C‐terminal tetramerization domain (Fig. 1). The available 3D structures from proteins of this family exhibit high similarity. However, so far only PAH has been crystallized as a full‐length protein, whereas models of the other enzymes have been assembled from various truncated protein constructs (Arturo *et al*. 2016). The N‐terminal regulatory domains of all AAAHs vary in length between ~100 and 160 residues, incorporating both an ACT domain (Zhang *et al*. 2014a; Arturo *et al*. 2016), and a highly flexible, perhaps even intrinsically disordered region (Nakashima *et al*. 2009; Zhang *et al*. 2014a; Louša *et al*. 2017). This has posed a major hurdle for crystallization of full‐length TH (Bezem *et al*. 2016). The catalytic domains of the AAAHs are highly conserved, consisting of ~330 amino acids (Flatmark and Stevens 1999) and a non‐covalently bound iron atom (Daubner *et al*. 1995). A 42‐residue long domain with a coiled‐coil motif present at the C‐terminus of the polypeptide facilitates oligomerization (Vrana *et al*. 1994; Goodwill *et al*. 1997; Tekin *et al*. 2014).

**Figure 1:**
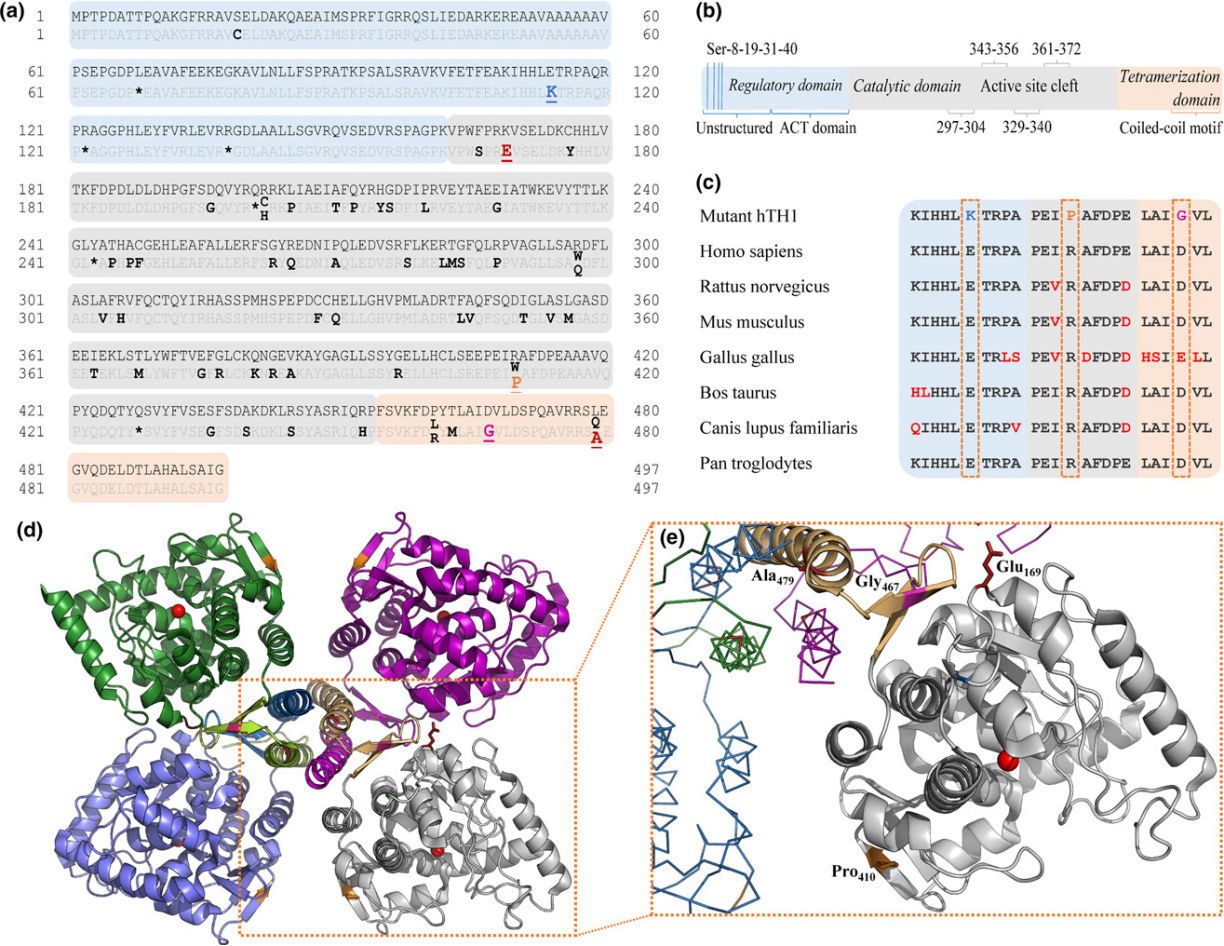
L‐dihydroxyphenylalanine (L‐DOPA)‐responsive dystonia (DRD)‐associated mutations of hTH1. UniProt reference sequence: P07101‐3. (a) All reported disease‐associated missense (bold) and nonsense (asterisk) mutations of the human TH1 gene with varying clinical manifestation (Fossbakk *et al*. 2014) until January 1st 2018 according to www.biopku.org/pnddb and more recent publications (Yan *et al*. 2017; Zhang *et al*. 2017). All mutations investigated in this study are shown underlined (including the experimental K169E/L479A double mutation). (b) A schematic look at the structure of the hTH1 subunit. (c) Amino acid sequence alignment shows that residues Glu‐114, Arg‐410 and Asp‐467 are highly conserved in mammals. (d) Structural overview of TH. The box highlights the catalytic (grey) and oligomerization (light orange) domains of one subunit and their relative positions within the native tetramer (PDB ID: 2XSN). Iron atoms coordinated within the active sites are illustrated with red spheres. Inlet (e) depicts the mutated side chains investigated in this work. Variant E114K is not shown as the N‐terminal 3‐D structure of TH is not yet resolved.

Tetrameric TH has an apparent molecular weight (MW) of ~240 kDa (Kumer and Vrana 1996; Haycock 2002), and the subunits interact through two distinct oligomerization interfaces, forming a dimer of dimers (Goodwill *et al*. 1997; Gordon *et al*. 2008) with P222 symmetry. While PAH has been observed to consist of a mixture of tetramers and dimers (Hufton *et al*. 1998; Kleppe *et al*. 1999), the other AAAHs are believed to exist exclusively as stable tetramers. The regulatory domain of PAH exists in an equilibrium of monomeric and dimeric species in solution, whereby binding of phenylalanine stabilizes the dimers (Zhang *et al*. 2014b). Such substrate‐induced multimerization has so far not been observed for either TPH1, TPH2 or TH, and it is considered to reflect the absence of a leucine heptad repeat in the C‐terminal helix in PAH; this has implications for the different physiological regulatory mechanisms governing these AAAHs (Arturo *et al*. 2016). However, for all of these enzymes, mutations that severely affect function and/or stability are often located in the active site or in the tetramerization domain (Goodwill *et al*. 1997; Fossbakk *et al*. 2014).

In humans, alternative splicing of pre‐mRNA (Kobayashi *et al*. 1988) in the central nervous system (CNS), peripheral sympathetic neurons and adrenal medulla (Nagatsu 1995) generate four isoforms of TH (hTH1‐4), of which hTH1 and hTH2 are the most abundant (Haycock 2002). The splice variants have different regulatory properties but only minor differences in catalytic constants (Haavik *et al*. 1991; Fossbakk *et al*. 2014).

Mutations in the hTH gene (chromosome 11p15.5) are primarily responsible for a rare autosomal recessive condition known as TH deficiency (THD), resulting in cerebral catecholamine deficiency. Despite the detailed characterization of many disease‐related TH variants, so far, no clear correlation between genotype and phenotype has been established (Knappskog *et al*. 1995; Ludecke *et al*. 1996; Fossbakk *et al*. 2014). THD is associated with several complex, clinically relevant phenotypes, such as autosomal recessive L‐DOPA‐responsive dystonia (DRD) – also known as Segawa's disease (Segawa *et al*. 1972) – and juvenile parkinsonism (Haavik *et al*. 2008) that encompass a range of debilitating but potentially treatable neurometabolic syndromes mainly affecting motoric functions (Willemsen *et al*. 2010). The possible role of TH in Parkinson's disease (PD) aetiology and treatment has been widely studied (Haavik and Toska 1998; Nakashima *et al*. 2013a,b; Nagatsu and Nagatsu 2016).

To explore whether DRD‐associated hTH1 variants could affect the oligomeric state of the enzyme, we characterized missense point mutants in exons 4 (E114K), 13 (R410P) and 14 (D467G) as well as the K169E/L479A artificial double mutant, that is reported to form a stable dimeric hTH1 species (Briggs *et al*. 2014). These mutations are located in the predicted regulatory, catalytic and tetramerization domains respectively (Fig. 1a, b and d). All three substituted amino acids – Glu‐114, Arg‐410 and Asp‐467 – are highly conserved in mammals (Fig. 1c). We found that some of these disease‐associated missense variants of hTH1 decrease its structural stability and introduce an abnormal equilibrium of tetrameric and dimeric species. We also report the discovery of stable and functionally active octameric species of the wild‐type enzyme.

## Experimental procedures

### Statistics

This study did not involve pre‐registration, randomization or blinding. Sample size was determined based on expected effects size derived from pilot experiments. Standard data processing pipelines were followed for the different experimental systems. Chemicals used in this study were purchased from Sigma unless otherwise stated.

### Mutagenesis, expression and purification of TH variants

Native human TH type 1 cDNAs were cloned into the pET3a expression vector (Haavik *et al*. 1991; Le *et al*. 1991). Coding sequences of the variants R410P (c.1322G>C), D467G (c.1493A>G), E114K (c.433G>A) and the double‐mutant K169E/L479A (c.598A>G; c.1528C>G; c.1529T>C) were created *via* site‐directed mutagenesis, as described (Fossbakk *et al*. 2014). All TH variants were expressed in *Escherichia coli* BL21(DE3) pLysS (Invitrogen, cat. no. C606003). Single colonies were inoculated into LB medium containing 50 μg/mL ampicillin and 34 μg/mL chloramphenicol and grown overnight at 30°C. Bacteria were grown at 37°C in 1 litre antibiotic‐containing LB medium until OD_600_ was 0.6. Expression of WT TH, E114K, R410P, D467G and K169E/L479A was induced with 1 mM isopropyl δ‐D‐thiogalactopyranoside at 25°C for 6 h at 180 rpm. Pellets were stored at −20°C until use. Upon thawing, cells were re‐suspended in lysis buffer consisting of 20 mM Tris‐HCl (pH 7.6), 1 mM EDTA, 1 mM dithiothreitol (DTT), 5% sucrose, 2 μM leupeptin, 1.5 mM benzamidine, 0.5 mM phenylmethylsulfonyl fluoride and one tablet cOmplete protease inhibitor cocktail (Roche Diagnostics GmbH, Mannheim, Germany). Cells were disrupted using a French pressure cell (GlenMills). The lysate supernatant was applied to the heparin Sepharose column pre‐equilibrated with 4× CV equilibration buffer (20 mM Tris‐HCl (pH 8.2), 150 mM NaCl, 1 mM EDTA, 1 mM DTT, 5% sucrose, 1 mM benzamidine, 0.25 mM phenylmethylsulfonyl fluoride). After binding, the column was washed with the same buffer before hTH1 was eluted with step‐wise increasing NaCl gradient *via* the combination of equilibration and elution buffers (the latter consisting of 20 mM Tris‐HCl (pH 8.2), 500 mM NaCl, 1 mM EDTA, 1 mM DTT and 5% sucrose). TH‐containing fractions were pooled and concentrated, using Amicon Ultra‐15 Centrifugal Filter Units with Ultracel‐50 membrane (Merck Millipore Ltd., Tullagreen Carrigtwohill, County Cork, Ireland). The proteins were further purified by size‐exclusion chromatography (SEC) using a Superdex 200 Increase 10/300 GL column (GE Healthcare, Chicago, IL, USA, cat. no. GE28‐9909‐44). The SEC buffer contained 20 mM Na‐HEPES (pH 7.4), 200 mM NaCl and 1 mM DTT. The purified proteins were concentrated and analysed by SDS‐PAGE on 10% precast gels (Bio‐Rad), prior to flash‐freezing in liquid nitrogen and storage at −80°C. The gels were stained with Coomassie Blue (InstantBlue™, Expedeon). Concentrations were determined by absorbance at 280 nm.

### Circular dichroism spectroscopy analysis of folding

Circular dichroism (CD) spectra of WT and mutants of hTH1 were recorded on a Jasco J‐810 spectropolarimeter in the wavelength range 205–270 nm. Measurements were carried out in triplicate with a protein concentration of 0.25 mg/mL in a 1‐mm quartz cuvette. The proteins were diluted into 10 mM HEPES (pH 7.4), 100 mM NaCl and 0.1 mM DTT. Signal from buffer was subtracted for each measurement.

### Analysis of thermal stability

A fluorescence‐based thermal stability assay (differential scanning fluorimetry, DSF) (Lo *et al*. 2004; Ericsson *et al*. 2006; Niesen *et al*. 2007) was performed using a LightCycler^®^480 II instrument (Roche). 10‐μL samples were analysed in 20 mM HEPES (pH 7.4) buffer with 200 mM NaCl and 1 mM DTT. Enzyme concentration was 0.1 mg/mL. SYPRO^®^ Orange was present in a final 1 : 1000 v/v dilution. The instrument was set to detect at wavelengths between 465 and 660 nm four times per temperature increments of 0.04°C/s from 20 to 99°C. Measurements were done in nine replicates. A 384‐well plate was used with an optical film (Roche).

Thermal denaturation was measured by CD spectroscopy at 222 nm from 20 to 95°C with a heating rate of 2°C/min. Samples were used at 0.25 mg/mL in 10 mM HEPES (pH 7.4), 100 mM NaCl and 0.1 mM DTT.

### Quaternary structure analysis

Analytical SEC coupled to multi‐angle static light scattering (MALS) was applied to determine the oligomeric states of purified hTH1 variants. Size exclusion was done using ÄKTA™Purifier FPLC system (GE Healthcare), which was coupled with a RefractoMax 520 module (ERC GmbH, Riemerling, Germany) for measuring refractive index for concentration determination, and a mini‐DAWN TREOS detector (Wyatt Technology) for measuring the light scattering. Each variant was diluted to 2 mg/mL, centrifuged at 16 000 *g* for 10 min at 4°C and filtered with 0.22 μm polyvinylidene difluoride centrifugal filters (Millipore). Two hundred microgram of the protein was applied onto the Superdex 200 Increase 10/300 GL column, pre‐equilibrated with 20 mM Na‐HEPES (pH 7.4), 200 mM NaCl and 1 mM DTT, at a flow rate of 0.4 mL/min. Astra software (Wyatt) was used for data analysis.

### Enzymatic activity assays and SEC‐enhanced activity profiles

In order to achieve high quality and reproducibility, all hTH1 samples were injected into identical reaction mixtures, containing 40 mM Na‐HEPES (pH 7.0), 0.5 mg/mL bovine serum albumin, 10 μM (NH_4_)_2_Fe(SO_4_)_2_ and 0.1 mg/mL catalase (Fossbakk *et al*. 2014); whereas Fe^2+^ was included in assays for maximum reaction velocity (Haavik *et al*. 1991). To investigate kinetic parameters, the substrate tyrosine (L‐Tyr) was present at 4–230 μM. The protein samples were diluted to 2–14 μM subunit concentration in the final reaction mixture and pre‐incubated on ice for 20 min. To facilitate reliable reaction product detection, mutants were present at higher concentrations than WT hTH1. Catalytic reactions were initiated by the addition of 200 μM BH_4_ with 2 mM DTT, subsequently incubated at 30°C in 5 min, and stopped by adding chilled absolute ethanol to 1 : 1 volume ratio. Formation of L‐DOPA was detected using high‐performance liquid chromatography (HPLC) with fluorometric detection (Haavik and Flatmark 1980). Kinetic parameters were determined at different concentrations of L‐Tyr by nonlinear regression analysis using the Michaelis–Menten equation in GraphPad Prism. All enzyme variants showed substrate inhibition at L‐Tyr concentrations above 80 μM. For comparison of kinetic values between different hTH1 species under physiological conditions, the calculations of *K*_m_ (L‐Tyr) and *V*_max_ were based on a substrate range of 0–60 μM L‐Tyr, where there was no apparent substrate inhibition for any of the variants, except for the artificial double mutant.

In order to assess the activity profile of different oligomerization states, 100 μg of hTH1 preparations were subjected to SEC on a Superdex 200 Increase 10/300 GL column pre‐equilibrated with 20 mM Na‐HEPES (pH 7.4), 200 mM NaCl and 1 mM DTT. The standard flowrate was 0.3 mL/min. Fractions were collected between 9.5 and 20 mL elution volumes. The eluate was monitored by absorbance at 280 nm and fractions were probed for activity and sampled for SDS‐PAGE.

### Size‐exclusion chromatography‐coupled small‐angle X‐ray scattering (SEC‐SAXS)

Small‐angle X‐ray scattering (SAXS) data were collected on the SWING (Small and Wide angle X‐ray ScatterING) beamline of SOLEIL synchrotron (Paris, France) in HPLC mode using a wavelength λ = 1.03 Å and a PCCD170170 (Aviex) detector at a 1.8 m distance, resulting in a momentum transfer q range of 0.01–0.62 Å^−1^ (q = 4π sin θλ^−1^; 2θ is the scattering angle). A SEC system was paired with SAXS data collection, enabling SAXS data collection from different oligomeric species (David and Pérez 2009). 50 μL of the proteins (hTH1‐WT at 1.5 and 7 mg/mL, hTH1 D467G at 3 mg/mL, hTH1 R410P at 2 mg/mL and hTH1 E114K at 5 mg/mL) were injected onto a ProSEC 300S (Agilent, Santa Clara, CA, USA) column pre‐equilibrated with the running buffer (20 mM HEPES (pH 7.4), 200 mM NaCl, 1 mM DTT). Buffer data were acquired at the beginning of the chromatogram, and sample data were collected in the peak areas. For each dataset, 255 frames were collected. Data reduction, *R*_g_ evaluation over elution profiles, data averaging and merging were performed using Foxtrot software.

Programs of the ATSAS (Konarev *et al*. 2006) package were used for data analysis and structure modelling. Distance distributions were analysed using GNOM (Svergun 1992).Three‐dimensional chain‐like *ab initio* models were created using GASBOR (Svergun *et al*. 2001). CORAL (Petoukhov *et al*. 2012) was used for hybrid model building, using the known structures of the catalytic [2XSN – (Muniz *et al*. TO BE Publ.)] and regulatory domains [2MDA – (Zhang *et al*. 2014a)]. Missing loops and termini were modelled, and the regulatory domain was allowed to move, whereas the catalytic domain tetramer was kept fixed. As good fits were obtained using this protocol, no additional degrees of freedom were considered necessary for reliable modelling.

### Negative‐stain transmission electron microscopy (TEM)

Samples for negative‐stain electron microscopy (EM) were prepared by applying 2 μL of the protein solution (at 0.15–0.7 mg/mL) on glow‐discharged carbon‐coated copper grids. After three short washes with Milli‐Q water, samples were stained with 1% uranyl formate solution and dried. Micrographs were recorded on an electron microscope (JEOL JEM‐1400) equipped with a LaB6 cathode and operated at 120 kV. Images were recorded with a 4096 × 4096 pixel CMOS TemCam‐F416 camera (TVIPS) at a nominal magnification of 60 000 and a corresponding pixel size of 2.29 Å under a defocus between 2.5 and 5.0 μm. Particles were picked from micrographs in e2boxer (Tang *et al*. 2007). Two‐dimensional classification and averaging were performed using Iterative Stable Alignment and Clustering (ISAC) (Yang *et al*. 2012). Three‐dimensional models were reconstructed using Validation of Individual Parameter Reproducibility (VIPER) implemented in SPARX (Hohn *et al*. 2007).

## Results

### Expression and purification of recombinant hTH1 variants

To investigate the oligomeric structure of hTH1, and the effects of DRD‐associated missense mutations, the WT and disease‐causing variants were expressed in *E. coli* and subjected to rapid, high‐resolution chromatographic separations in the presence of protease inhibitors (Figure S1, Fig. S2b). A previously described artificial double mutant of hTH1 that forms stable dimers was used as a positive control for the dimeric species. This mutant was originally described as K170E/L480A (Briggs *et al*. 2014); however, based on the consensus sequence of hTH1 (NM_000360.3), the correct identifier should be K169E/L479A. All enzymes, including the disease‐related variants R410P (Haugarvoll and Bindoff 2011), D467G (Furukawa *et al*. 2001) and E114K (first characterized here) were expressed in the absence of N‐terminal fusion tags. The rationale was to eliminate their influence on protein folding, as well as to prevent contamination from metal affinity resins, which is known to inhibit TH through replacing iron in the active site (Schünemann *et al*. 1999; Bezem *et al*. 2016). After affinity purification on heparin‐Sepharose the protein yields were highest for the WT enzyme (29.2 ± 2.8 mg/L culture, *n* = 3), compared to 20.5 ± 3.8 mg/L, *n* = 3 for D467G, 9.77 ± 0.1 mg/L, *n* = 3 for E114K and 6.3 ± 0.6 mg/L, *n* = 3 for R410P (Fig. S2a). This could indicate that the mutant enzymes were produced at lower levels or were subject to partial degradation or precipitation during expression. Although the exon 4 mutation was not expected to have major influence on the stability of the enzyme, the protein yield for E114K remained at only 33% of that of the WT enzyme, even below the levels of D467G (70%) which was predicted to be a structurally more severe mutation. As shown in Fig. S1c and d, all hTH1 variants were obtained in high purity. Based on comparison with molecular weight standards, WT hTH1 was always recovered as a mixture of apparent octameric (14.4 ± 3.7%, SD, *n* = 7) and tetrameric species (85.6 ± 3.7%, SD, *n* = 7), whereas missense variants R410P and D467G were mixtures of tetramers and dimers, together with variable amounts of high‐molecular weight aggregates and partially degraded enzyme (Figure S1b). In contrast, E114K eluted as a tetramer preceded by a small amount of aggregated protein. The different oligomeric species of hTH1 were collected during gel filtration (Fig. S1b), concentrated and later subjected to activity measurements (Table 1; Fig. 3; Fig. S5) and biophysical characterization.

**Table 1:** Enzymatic activity. *V*_max_ and *K*_m_ (L‐Tyr) values shown here represent the best‐fit values within 95% confidence interval ± standard errors (*n* = 3 for each substrate concentration)

hTH1	Tertiary assembly	%	V_max_ (nmol/min/mg)	K_m_ (μM)	*R* ^2^
WT	T	100	2685 ± 162.2	33.7 ± 4.3	0.983
	O	66.5 ± 2.2	1786 ± 58.8	21.4 ± 1.9	0.925
E114K	T	89.6 ± 3.6	2405 ± 139.6	30.2 ± 3.9	0.982
R410P	T>D	19.1 ± 0.8	513.2 ± 22.4	33.5 ± 3.4	0.985
D467G	T>D	7.4 ± 0.3	199.2 ± 6.9	22.2 ± 1.9	0.987
	D>T	5.1 ± 0.1	136.3 ± 3.5	24.3 ± 1.5	0.994
K169E/L479A	D	2.8 ± 0.2	75.9 ± 3.6	7.1 ± 1.0	0.953

‘%’ represents the percentage of enzymatic activity (*V*_max_) compared to the WT enzyme.

### Quaternary structure analysis

**Figure 2:**
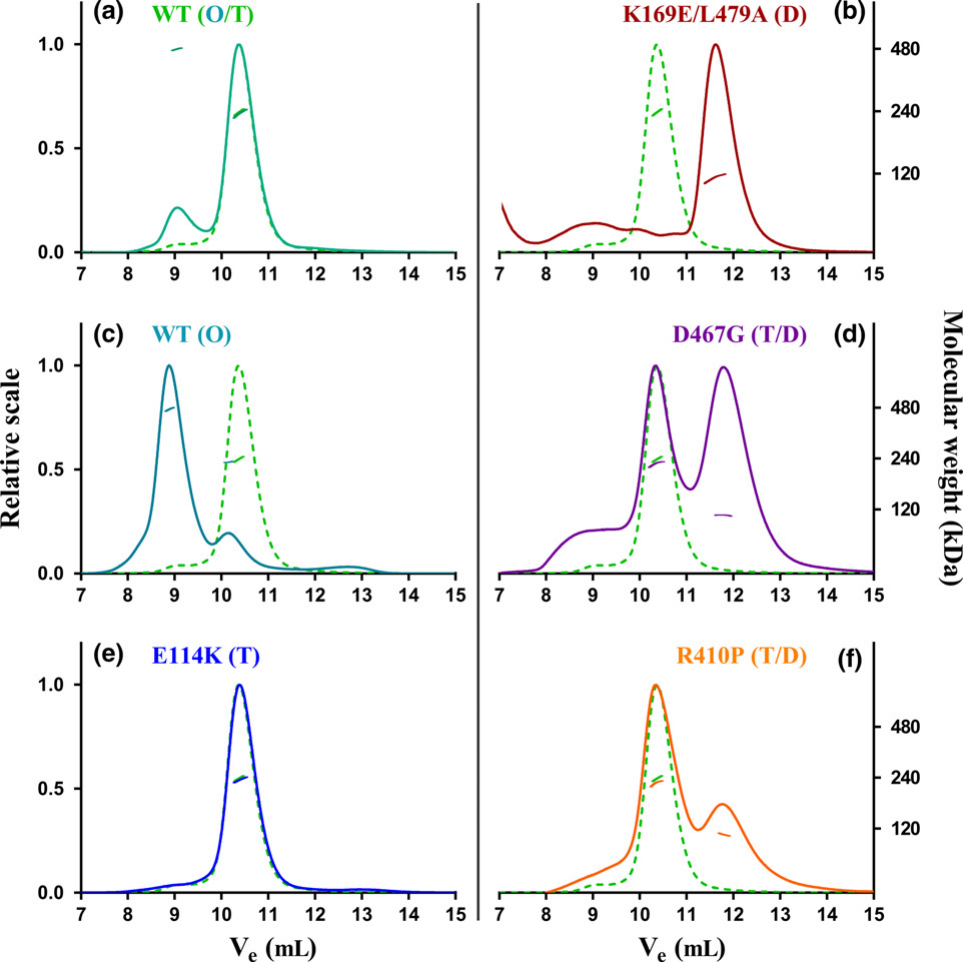
SEC‐MALS measurements of WT and disease‐causing hTH1 variants. This figure illustrates the different oligomeric forms observed, where ‘O’ stands for octameric, ‘T’ for tetrameric and ‘D’ for dimeric species. For easy comparison, WT (T) (green dashed line) is shown alongside each variant; the mixed protein batch of WT (O/T) (a), the octameric WT (O) (c); the E114K (T) variant (e), the artificial double‐mutant K169E/L479A (D) (b) and finally the mixed oligomeric states of variants D467G (T/D) (d) and R410P (T/D) (f).

Absolute molecular weight (MW) determination using SEC‐MALS confirmed the tertiary and quaternary structure of the different species (Fig. 2). MWs ascertained for the WT tetramer (T) and WT octamer (O) (Fig. 2a and c) were in correspondence to the theoretical values of the WT hTH1 tetramer (~240 kDa) and the hypothetical octamer (~480 kDa). The WT (O) elution profile (Fig. 2c) indicated that separation of octameric and tetrameric fractions during the last step of purification (Fig. S1b) was not fully achieved. E114K (T) (Fig. 2e) behaved identically to the WT (T), whereas mutants R410P (T>D) and D467G (T>D) displayed variable dimeric content in addition to their respective tetrameric fractions (Fig. 2f and d). The dimeric species of these two variants appeared to be ~3.4% smaller when compared with the positive control, K169E/L479A (D) (Fig. 2b). Calculated MW values are summarized in Table 3.

### Enzyme kinetics of the different oligomeric forms of hTH1

The exon 4 mutation E114K had modest effects on both *V*_max_ and *K*_m_ for the substrate L‐Tyr, as the maximum velocity reached nearly 90% of that of the WT hTH1 (T). The specific activity of the octameric WT hTH1 was 66.5% of the tetramer, but the *K*_m_ for tyrosine was also lower, resulting in identical specificity constants (*k*_cat_/*K*_m_ ratio) for these species. Previously, R410P has been reported to be unstable, with no detectable enzyme activity, and less than 6% residual activity was attributed to the D467G variant (Fossbakk *et al*. 2014). In this study, probably due to optimized purification conditions, both mutants displayed considerable residual activity. In enzyme fractions mainly containing the tetrameric forms of these enzymes (T>D), the residual activities of R410P and D467G were 19.1% and 7.4% compared to the tetrameric WT. Although the dimer‐enriched fractions of D467G (D>T) displayed lower specific activity (5.1%), it was still higher than the entirely dimeric K169E/L479A (D) (2.8%). The presence of some tetrameric TH in the D467G (D>T) sample could be in part responsible for its higher catalytic activity. The kinetic parameters are summarized in Table 1. Similar substrate inhibition was evident at L‐Tyr concentrations above 80 μM for both the mutants and WT hTH1 (Fig. S5).

**Figure 3:**
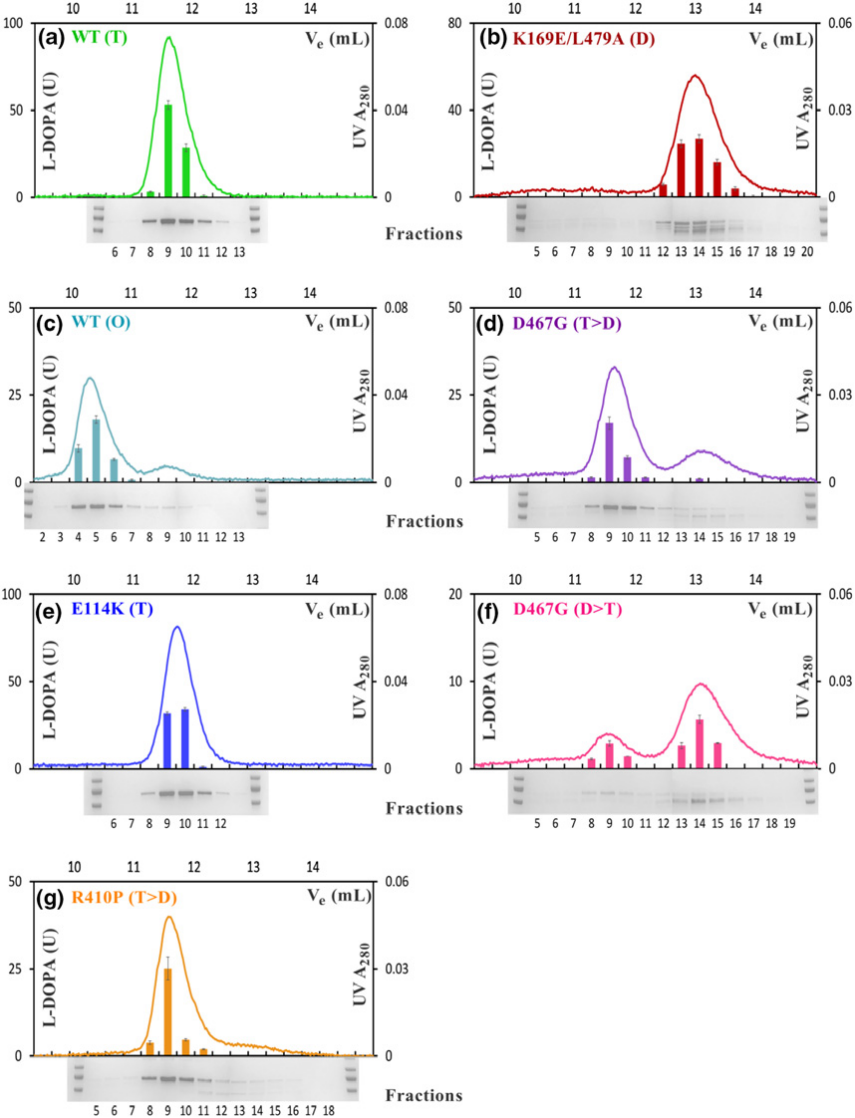
Study of quaternary structure with enzymatic activity profiles of recombinant hTH1 variants. SEC data for the hTH1 variants are presented as WT (T) & (O) (a and c), E114K (T) (e), R410P (T>D) (g), K169E/L479A (D) (b) and lastly D467G (T>D) & (D>T) (d and f), which exhibits a dynamic equilibrium between tetrameric and dimeric species. This graphic combines measurements of UV absorbance at 280 nm (right Y axis, line), average activity readings (*n* = 3 separate activity measurements, left Y axis, columns), expressed as L‐DOPA production in units (based on absorbance measured between wavelengths 281–314 nm) with complementary SDS‐PAGE analysis of the eluate collected in 0.3 mL fractions (X axes). A pre‐stained molecular weight standard (PageRuler^®^) was present during each electrophoresis (first and last lanes, 100, 70 and 55 kDa standards are shown). One unit (U) of TH activity is defined as 20 picomol L‐DOPA produced in 100 μL reaction volume at 30°C in 5 min. The error bars correspond to the standard error of the average reaction velocity calculated from three parallel activity assays.

As demonstrated in Fig. 3, octameric, tetrameric and dimeric hTH1 species showed closely corresponding activity and protein elution profiles. R410P displayed activity only in its tetrameric fraction, whereas D467G appeared to consist of active tetrameric and dimeric species. SDS‐PAGE analyses of the corresponding protein fractions showed that the WT hTH1 (T, O) and E114K migrated as single bands, whereas the dimeric fractions of C‐terminal mutants R410P, D467G and K169E/L479A were partially degraded. Thus, it is possible that the catalytic activity could be in part attributed to partially degraded hTH1. Indeed, tryptic peptide mapping followed by mass spectrometry confirmed partial C‐terminal degradation for R410P, D467G and K169E/L479A variants (Table S1). However, the presence of non‐degraded enzyme within all dimeric fractions suggests that changes, for example misfolding caused by the mutation might be responsible for disrupting the quaternary structure, and making the C‐terminus exposed and available for proteolytic degradation.

### Folding and thermal stability of hTH1

**Figure 4:**
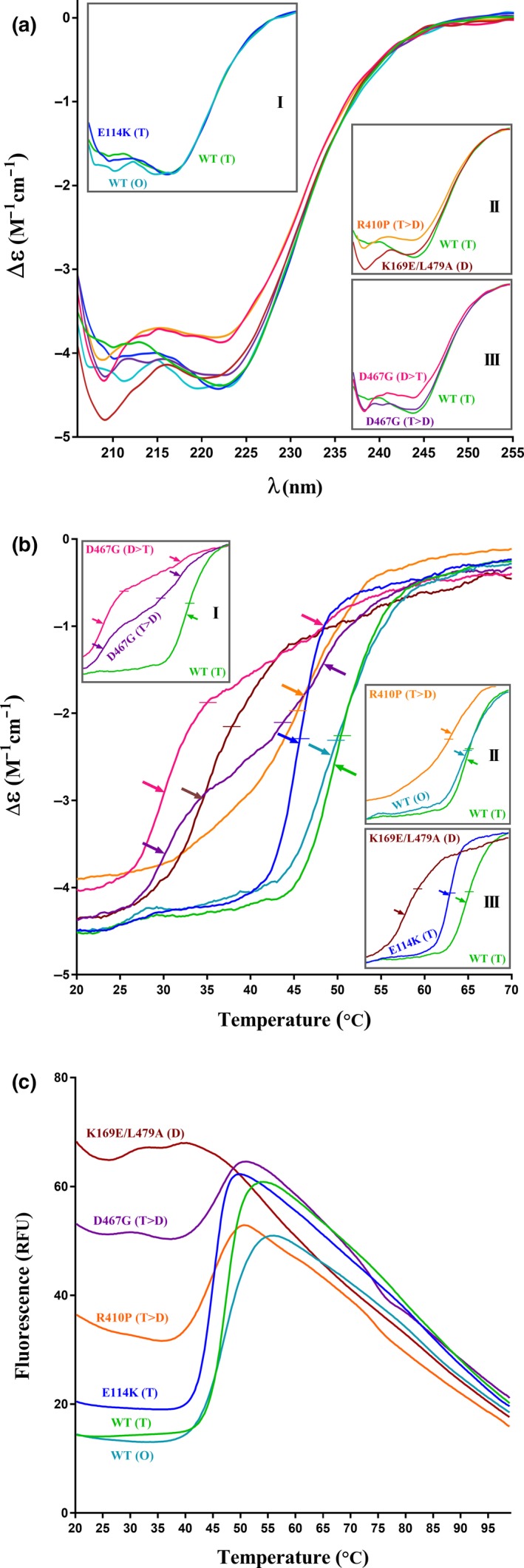
CD spectra and thermal stability of WT and mutant proteins. (a) Spectra were measured between 205 and 270 nm (*n *=* *3 replicates of each sample). The insets highlight key differences in helical content between the WT and mutants. (b) Thermal denaturation was measured at 222 nm over increasing temperatures as changes in CD signal. The top left inset presents the difference in thermal stability between tetramer‐enriched (T>D) and dimer‐enriched (D>T) fractions of D467G, indicating the instability of the dimeric form. *T*_m_ (temperature at which the protein is half denatured) is shown with horizontal lines, whereas arrows highlight the first derivatives. For respective *T*_m_ values, refer to the Table 2. (c) Differential scanning fluorimetry (DSF) analysis of thermal stability. The averaged *T*_m_ values (*n* = 9 replicates of each sample) follow the trend that was drawn from the CD data (b).

The secondary structure and thermal stability of the different species were examined using DSF and CD spectroscopy. Native WT hTH1 has been reported to have a CD spectrum with two characteristic minima at ~208 and 220 nm and a local maximum at ~215 nm (Kleppe and Haavik 2004). This was also observed here for WT and E114K enzymes, although with slightly shifted maxima (Fig. 4a inset I). In contrast, the spectra of the R410P, D467G and K169E/L479A variants were markedly different, possibly because of disturbed coiled‐coil structures in the C‐terminal tetramerization domain, leading to a decrease in the signal intensity at ~220 nm, whereas the peak at ~208 nm was stronger (Fig. 4a inset II). Indeed, tetramer‐ and dimer‐enriched fractions of D467G displayed clear differences, probably also reflecting on the extent of degradation of these fractions (Fig. 4a inset III).

Previous biophysical characterizations have shown decreased thermal stability *in vitro* for most disease‐related TH missense variants (Haavik *et al*. 2008; Fossbakk *et al*. 2014). Both CD spectroscopy and DSF showed a clear decrease in thermal stability between the WT hTH1, whether as tetramers or octamers, and all mutants investigated in this work (Fig. 4b and c; Table 2). The melting curves obtained by CD spectroscopy displayed further differences among the variants: E114K (T) had a similar profile to that of the WT hTH1 (T) but with decreased thermal stability, whereas dimer‐enriched D467G (D>T) and K169E/L479A (D) showed the lowest stability and a less pronounced CD signal transition. Variants R410P (T>D) and D467G (T>D) that are prone to occupy two distinct oligomerization states appeared shifted, displaying a complex transition during unfolding. The top left inset in Fig. 4b emphasizes the difference between fractions containing primarily dimeric or tetrameric species of D467G.

**Table 2:**
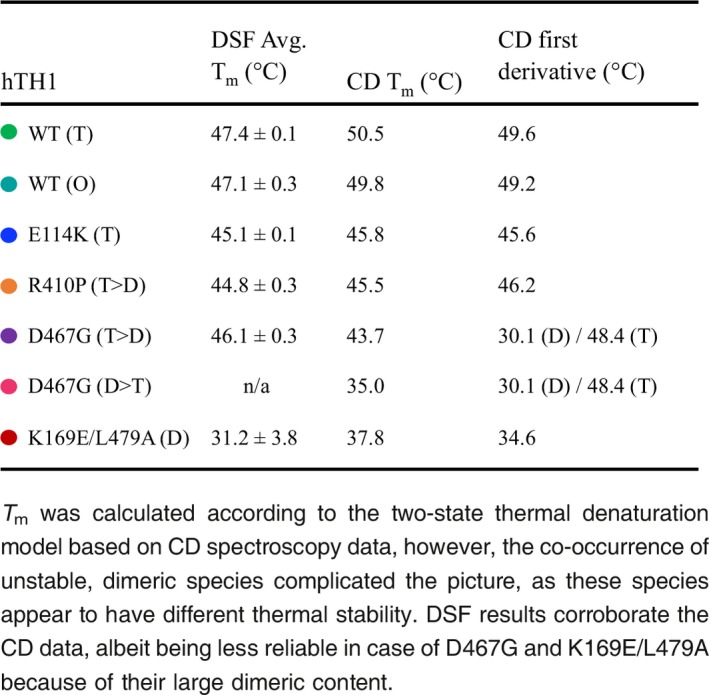
Melting temperatures (*T*_m_) of hTH1 variants

DSF showed that the larger the dimeric fraction was within the enzyme pools of mutants; the higher initial fluorescent signal was detected for the respective samples. At the molecular level, this translates to an increased accessibility of hydrophobic areas for the binding of SYPRO Orange dye on the dimer (Fig. 4c). Melting point measurements for the double mutant as well as R410P (T>D) and D467G (T>D) suffered from high initial signal primarily because of the presence of more exposed dimeric content and thus were less reliable using this assessment. Our findings summarized in Table 2 show differences in the melting points obtained *via* the two methods. It is important to point out that as the two approaches measure different indicators for protein denaturation – notably, the unfolding of secondary structures versus access to hydrophobic parts through the motions of tertiary structures, respectively. Therefore, the respective melting temperatures (*T*_m_) can be expected to differ.

### Structural studies confirm that mutations alter hTH1 conformational and oligomeric properties

**Figure 5:**
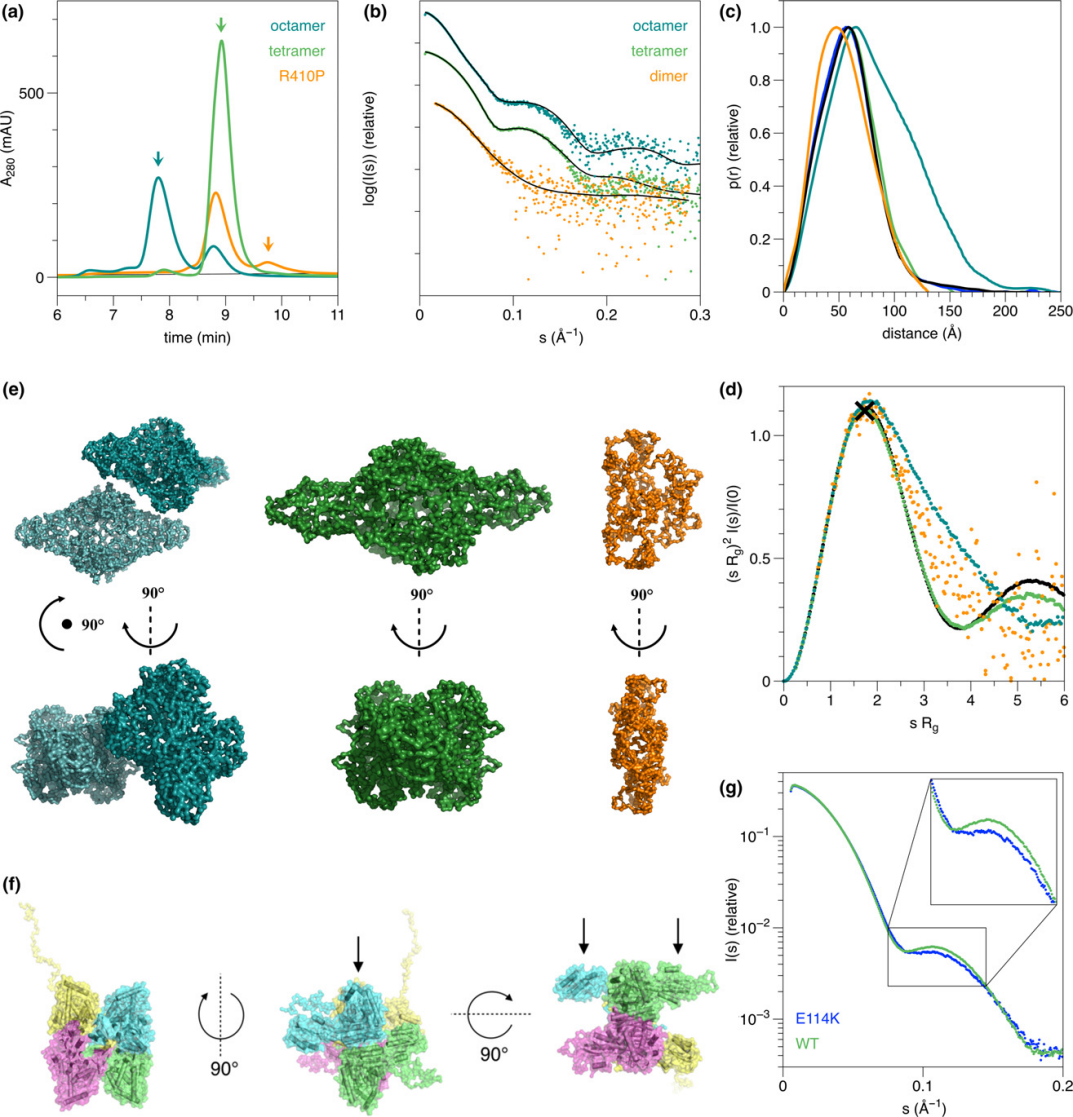
SAXS‐based modelling of hTH1 structure in solution and the effects of missense mutations therein. (a) SEC profiles during the online SEC‐SAXS experiment. Three samples are shown: tetrameric hTH1 (green), octameric hTH1 (cyan) and the R410P variant (orange). The arrows indicate positions of peaks shown in the following panel. (b) SAXS patterns from octameric, tetrameric and dimeric species of hTH1. The black lines represent fits of the chain‐like ab initio models, shown in panel E, for the different species. (c) Distance distribution functions for the three patterns from panel B, plus another measurement for tetrameric hTH1 (black) and the tetramer peak from E114K (blue), to show reproducibility. The tetramer curves superpose so well that they cannot be distinguished from one another. (d) Dimensionless Kratky plot. The black cross marks the position of the maximum (x=√3, y = 1.1) for a globular perfectly folded particle. (e) Chain‐like models for the hTH1 octamer (cyan), tetramer (green) and the R410P dimer (orange) are shown in two orientations similarly as in the next panel, middle (top) and left (bottom). (f) Hybrid modelling of the hTH1 tetramer; three orientations separated by 90° rotations are shown. Left: view along the tetramerization axis; middle: the catalytic domains arrange as a plane in the middle (arrow); right: the regulatory domains are not forming dimers in the models, but stay separated on both sides of the central planar arrangement. (g) The tetrameric forms of hTH1 and the E114K variant have different conformations, as highlighted by clear differences in the middle region of the scattering patterns.

SEC‐SAXS was used to obtain low‐resolution 3D structures of WT and mutant hTH1 enzymes in solution. This combination of SEC with SAXS enables the removal of aggregates, separation of different oligomeric states and the accurate acquisition of scattering curves from the SEC peak that contains the sample of interest (David and Pérez 2009). SEC‐SAXS confirmed the presence of different oligomeric states of hTH1. A tetrameric form was present each in each sample, with a radius of gyration R_g_ ≈ 45 Å. The maximum interatomic distance *D*_max_ was ~190 Å for WT, whereas the mutants exhibited slightly smaller values (Table 3). Judging from the distance distribution, the differences between the different protein variants were minor. The octameric hTH1 had clearly larger dimensions and volume than the tetrameric species. Both the distance distribution function (Fig. 5c and e) and modelling (see below) support these observations. The dimensionless Kratky plot (Fig 5d) indicates that the hTH1 tetramer and octamer are in a highly folded state, but the dimeric form of the R410P variant shows more flexibility.

**Table 3:** Molecular weight of hTH1 variants based on MALS, as well as SAXS data collection, processing and analysis. *R*_g_ was obtained from the Guinier plot, and *D*_max_ and the Porod volume were estimated using GNOM (Svergun 1992)

hTH1	Tertiary assembly	MW (MALS) (kDa)	*R*_g_ (Å)	*D*_max_ (Å)	Porod volume estimate (nm^3^)
WT	T	236.4 (± 0.2%)	45.2 ± 0.7	190	446
	O	481.8 (± 0.5%)	64.1 ± 0.4	250	919
E114K	T	235.3 (± 0.3%)	44.5 ± 0.2	170	422
R410P	T	225.4 (± 0.3%)	45.2 ± 0.3	160	429
	D	111.7 (± 0.2%)	41.9 ± 0.5	130	278
D467G	T	225.7 (± 0.1%)	45.2 ± 3.7	150	439

The chain‐like *ab initio* model of tetrameric hTH1 presents an elongated assembly, and its volume is appropriate for a tetramer. Similar models, with somewhat larger dimensions, were seen before, when SAXS was carried out in batch mode (Bezem *et al*. 2016). This highlights the advantages of using SEC‐SAXS for proteins prone to form higher order oligomers. The octameric form of hTH1 could be modelled well by a simple rigid body refinement of two chain‐like models of the hTH1 tetramer. The tetramers appeared to be associated side‐by‐side. The dimeric form for the R410P variant was also modelled, and a structure of smaller dimensions that the tetramer was obtained, whose shape is reminiscent of one half of the full tetramer (Fig. 5e). This confirms the presence of a stable dimeric form of hTH1 in the mutant preparation. The structure of the dimeric form appears relatively looser than those of the tetrameric assemblies; this is in line with the Kratky plot.

Hybrid models based on known structures of the catalytic and regulatory domains, the known tetrameric assembly of the catalytic domain, and missing loop building were used to obtain a better understanding of flexibility and domain organization in hTH1. The tetrameric arrangement, with P222 symmetry, of the catalytic and oligomerization domains was kept fixed, whereas the N‐terminal tail and the regulatory domain were built without symmetry restraints. The regulatory domain for each monomer was also modelled without restraints. The ability to fit the models well to the experimental SAXS data suggests rigidity of the core hTH1 assembly. The catalytic and tetramerization domains form a planar core of the hTH1 structure (Fig. 5f, arrow). The regulatory domains are all positioned close to the respective catalytic domain; however, no formation of regulatory domain dimers is observed (Fig. 5f, arrows). One of the N termini is always extended in the models, reflecting the flexibility of this region. The data are average over different conformations, and this observation in single models trying to represent reality simply highlights the presence of elongated N termini in the conformational ensemble.

For the hTH1 mutants, *R*_g_ values similar to the WT protein were observed, whereas *D*_max_ appeared smaller (Table 3). Details of the scattering curves are also clearly different (Fig. 5g), indicating true conformational differences. Because of the higher concentration, the variant E114K (T) was considered more reliable for analysis, although the overall shape and derived parameters for R410P (T) are essentially identical. The data are compatible with a scenario, in which the mutant hTH1 tetramers may be less flexible/more compact than the WT tetramer, and this in turn may prevent functionally important conformational changes during the catalytic cycle, rendering the enzyme less active. The same variants, on the other hand, presented additionally active dimeric species, indicating defects in oligomeric state. The D467G variant was prone to aggregation, and even after SEC‐SAXS, some aggregation was evident in the X‐ray beam.

**Figure 6:**
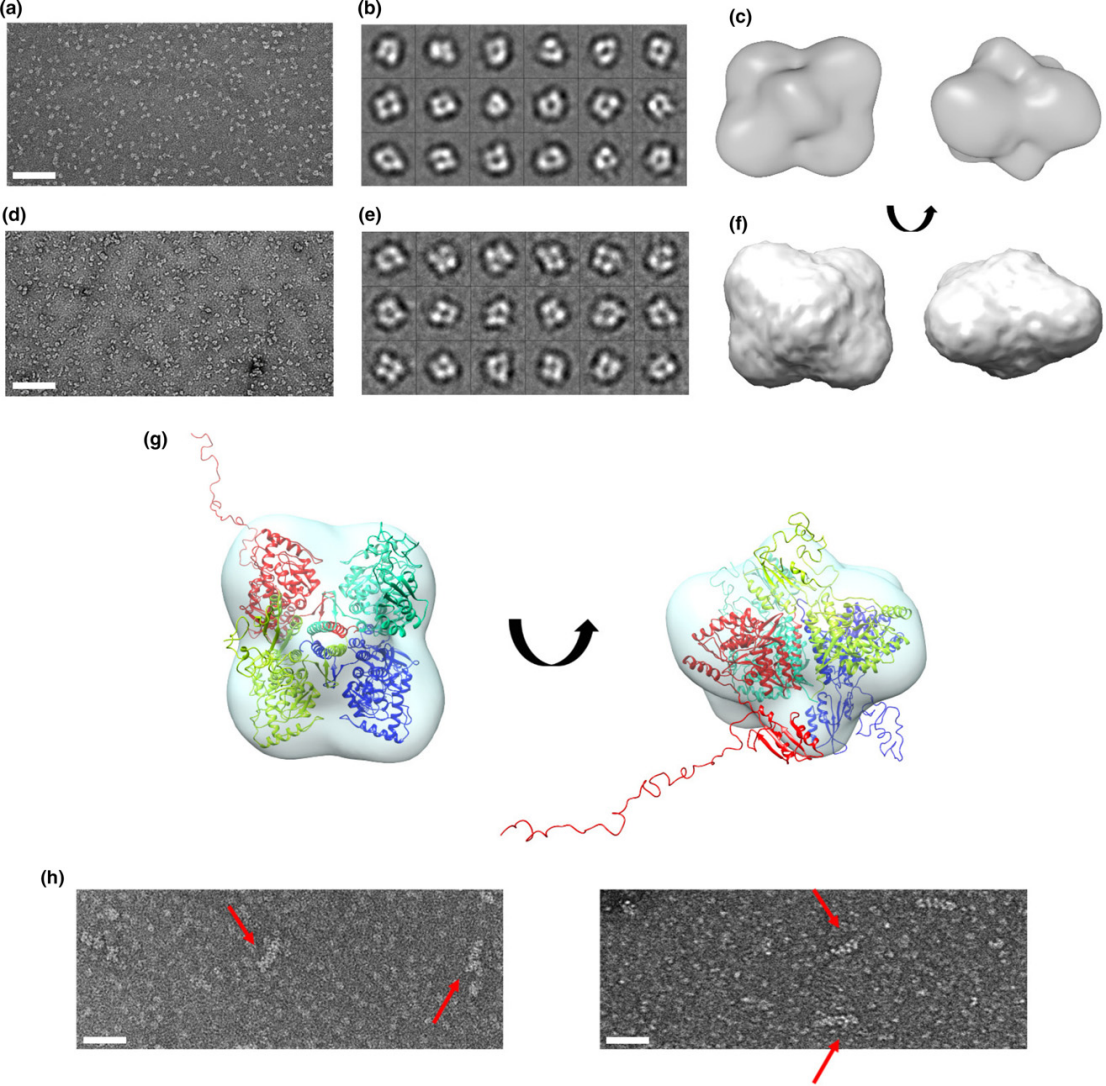
Transmission electron microscopy (TEM) analysis of WT (T & O) and R410P (T) of human TH1. (a and d) Representative TEM micrographs, (b and e) Representative 2D‐class averages, (c and f) 3D‐models in two different views of WT (T) and R410P (T) respectively. (g) Superposition of the crystallographic model of the catalytic and tetramerization domain onto the TEM envelope of WT hTH1 (T). (h) TEM analysis of WT hTH1 (o). Two representative micrographs are shown, displaying mostly particles of larger diameter (suspected octamers) and filaments. The higher order oligomers assembled in the form of long chains are indicated by red arrows. The scales represent 100 nm.

Aliquots of hTH1 were negatively stained and observed under an electron microscope. The micrographs mainly visualized single monodisperse protein particles in two preferential orientations consistent with side and top views. A total of 10 500 and 9300 particles were selected and processed for the best behaving constructs hTH1 WT (T) and hTH1 R410P (T) respectively (Fig. 6). Two‐dimensional class averages calculated for the WT (T) and R410P (T) displayed characteristic top views indicating the presence of four subunits and characteristic side‐views in the shape of tube‐like structures. Comparing the SAXS hybrid model with a model obtained from single‐particle TEM experiments, a strong similarity is observed (Figs 5f and 6g).

The octameric species (modelled in Fig. 5e) are presumably formed by the side‐by‐side association of two homotetramers in solution. This species is also enzymatically active, and interestingly, when WT (O) was studied by TEM, it mainly presented larger diameter particles together with a small amount of filament‐like structures (Fig. 6h). These filaments seem to only represent a minor, apparently aggregated fraction of the total enzyme pool, probably corresponding to the small shoulder appearing in front of the octameric main peak (Figs 2c and 3c). It is likely that these assemblies were formed by the same side‐by‐side mechanism between tetramers as the observed octamer (Fig. S6). Nevertheless, owing to this inhomogeneity of the octamer‐enriched WT (O) sample (Fig. S1b and d), constructing a TEM‐based model analogous to that of WT (T) and R410P (T) has proven to be difficult. Together, these data indicate that, in the absence of a crystal structure of full‐length hTH1, the models presented based on SAXS and EM are reliable and represent the true conformation of the hTH1 tetramer.

## Discussion

Here, we report the first detailed characterization of the oligomeric structure of TH in solution. Mammalian TH has previously been expressed in *E. coli* with or without fusion partners. While fusion tags facilitate protein purification, such artificial sequences can also interfere with proper protein folding and, as shown for TH, leakage of metal ions from the metal affinity resins used for affinity chromatography inactivates the enzyme by blocking the incorporation of iron into its active site (Schünemann *et al*. 1999; Bezem *et al*. 2016). We expressed the full‐length native hTH1 enzyme variants without fusion tags, resulting in highly active and stable enzymes (Table 1). Peptide mapping showed that both WT and disease‐associated hTH1 variants were recovered as full‐length proteins, with completely intact C‐terminal amino sequences, and N‐terminal sequences from amino acids 13 or 17 (Table S1). However, it remains unclear whether the first 12 N‐terminal residues were also intact, as conclusively identified peptides were not recovered for this sequence.

We demonstrate that wild‐type human TH 1 is present as a mixture of stable octameric and tetrameric species with comparable catalytic activities. The octameric species of TH has previously not been reported. This is likely because of insufficient chromatographic resolution in previous studies. However, on closer inspection of SEC‐coupled activity measurements of freshly processed animal tissue lysates (Fig. S3b), or TH expressed as recombinant proteins, there have been indications of some higher‐MW forms, possibly corresponding to octameric species (Briggs *et al*. 2014). Furthermore, PAH from the same hydroxylase family has been shown to form octamers (Kleppe *et al*. 1999). The origin of the observed octameric hTH1 assemblies is not known, and SDS‐PAGE analyses performed in the presence or absence of dithiothreitol (DTT) provided no explanation either (Fig. S4). As the emergence of these species was unaffected by reducing agents like DTT, which was present throughout the purification process, as well as within the storage buffer, they appear not to be caused by the formation of intermolecular disulphide bonds. Alternatively, the N‐terminal ACT domains could be involved in these intermolecular complexes (Zhang *et al*. 2014a; Fitzpatrick 2015). All the aromatic amino acid hydroxylases contain such domains, that are named after their occurrence in several allosterically regulated oligomeric enzymes (Lang *et al*. 2014). The ACT domains are involved in subunit interactions and oligomerization and are found in dimeric, tetrameric, as well as octameric enzymes (Xu and Grant 2014). Even higher order assemblies involving the ACT domains have also been described (Xu and Grant 2014). For TH, we mainly observed tetrameric and octameric species, but EM also revealed small amounts of filamentous structures (Fig. 6h), compatible with chains of tetramers, possibly linked via ACT domains (Fig. S6). It is yet unknown whether the octameric and filamentous species are found *in vivo* during, for example protein production, transport or turnover, or are involved in pathological processes. Because of the lack of access to brain tissue from carriers of TH mutations or healthy controls, we cannot conclusively extrapolate our findings to the intact human brain. However, we performed high‐resolution SEC‐coupled enzyme activity assays on freshly prepared lysates of bovine adrenal medulla, a tissue source rich in TH that is often used for studies of catecholamine biosynthesis and TH regulation, and found indications of higher order oligomeric species of TH in the activity profile, possessing approx. 15% of total TH activity (Fig. S3b). In conclusion, the data obtained from this experiment using mammalian tissue lysates support our findings that came from employing a prokaryotic system for protein production. Nevertheless, it also remains unclear how the oligomeric structure of TH might be influenced by its binding partners, including possible ligand binding to the ACT‐domain, as reported for phenylalanine binding to PAH (Arturo *et al*. 2016). It is well established that TH is activated and stabilized by binding to 14‐3‐3 proteins (Kleppe *et al*. 2014) and it has also been suggested that TH might be part of macromolecular assemblies with multiple protein components that are involved in dopamine synthesis and transport (Parra *et al*. 2016). However, more recent imaging data are not compatible with such multi‐protein complexes, at least in dopaminergic neurons (Rahbek‐Clemmensen *et al*. 2017).

We also report a detailed structural and functional characterization of two known (R410P, D467G), and a recently found (E114K) THD‐associated variants. The former mutations are associated with more severe phenotypes, whereas the latter is associated with mild THD‐associated symptoms. Evolutionary analysis on the peptide sequence shows that all three residues affected by missense mutations are highly conserved in mammals (Fig. 1b), which relates to their importance for the structure and enzyme function of TH (Nagatsu and Ichinose 1991). To predict the severity of the amino acid substitutions, we performed *in silico* analysis using Polyphen‐2 (http://genetics.bwh.harvard.edu/pph2). The R410P mutation was predicted to be the most likely damaging (score: 1.000; sensitivity: 0.00; specificity: 1.00), mainly because of the disruptive nature of proline in secondary structural elements (Fig. 1d). Interestingly, this was followed by E114K (score: 0.999; sensitivity: 0.14; specificity: 0.99), whereas the D467G substitution was predicted to be probably damaging (score: 0.935; sensitivity: 0.80; specificity: 0.94). However, in contrast to the *in silico* predictions, activity assays showed that the D467G mutation was indeed the most damaging, with only 5‐7% retained activity compared to the WT (Table 1). In contrast, E114K maintained 90% residual activity. Thermal stability measurements also corroborated the functional analyses. This illustrates the need to perform experimental validation to complement *in silico* mutation severity prediction.

Proper oligomeric assembly of TH is essential for its physiological function. As shown by SEC, combined with MALS, SAXS and electron microscopy; active, full‐length hTH1 can exist as dimers, tetramers and octamers. Partially active dimeric species of the disease‐associated variant D467G were also present in solution (Fig. 3d and f). D467G is hypothesized to be in a dynamic equilibrium between tetrameric and dimeric species. Thus, the protein peaks corresponding to tetrameric and dimeric fractions were concentrated separately, but in contrast to the WT, upon additional size‐exclusion chromatography steps, mixtures of tetramers and dimers were always observed. A structural explanation could be that the Asp – Gly exchange results in excess conformational freedom *via* the loss of a critical H‐bond and thus decreased stability of the D467G quaternary structure (Fig. 1d). The R410P mutation affected the protein structure differently. It had a low abundance of dimers (Fig. 2f, Fig. 5a) with no detectable enzyme activity (Fig. 3g) and an increased susceptibility to proteolysis (Fig. S1c, Fig. 3g). The proline substitution towards the oligomerization domain (Fig. 1d) is expected to disrupt a local beta sheet and to introduce conformational rigidity. Consequently, this structural instability may lead to misfolding, potentially irreversible disassembly and proteolysis. Both R410P and D467G had significantly lower melting points than the WT. Indeed, most disease‐associated TH variants have decreased thermal stability *in vitro* (Royo *et al*. 2005; Fossbakk *et al*. 2014). Complicating this general observation, the denaturation curve of R410P and D467G indicated the presence of more than two states; a dimeric, very unstable fraction, together with a tetrameric fraction that shifts towards dimeric conformational states while unfolding. In essence, it is apparent that these two mutations affect enzyme function primarily through interfering with quaternary assembly and stability, possibly leading to increased cellular turnover *via* misfolding‐induced degradation (Winge *et al*. 2007).

It is not clear what the exact functional impact of E114K may be *in vivo*; however, SAXS data have revealed clear conformational differences, in which a less flexible/more compact mutant tetramer may be prevented from going through functionally important conformational changes during the catalytic cycle, hindering its physiological function. TH is tightly regulated through its N‐terminal regulatory domain (Kumer and Vrana 1996; Ramsey and Fitzpatrick 1998; Nakashima *et al*. 2009); therefore, these conformational changes are likely related to this intricate control over TH activity, including the stabilizing protein‐protein interactions with 14‐3‐3 (Daubner *et al*. 2011; Fitzpatrick 2015; Waløen *et al*. 2017). Consequently, in the absence of major changes in the protein structure, altered regulatory properties may also play a role in the pathophysiology of THD‐associated mutations.

## Concluding remarks

The purpose of this work was to study the quaternary structure of TH and the effects of disease‐associated mutations on hTH1 structure and activity. For the first time, we show that DRD‐associated human mutations may interfere with the normal oligomeric status of hTH1. The observations of catalytically active octameric and dimeric forms of hTH1 add to our understanding of the complex nature of this important regulatory enzyme and warrant further investigations on how such species may be involved in physiological and pathological processes. Increased knowledge regarding the genotype‐phenotype correlations on the molecular level might also open up new treatment options for subgroups of patients with destabilizing TH mutations. For instance, unstable missense variants might be targets for pharmacological intervention by employing designated pharmacological chaperones, aimed to re‐establish the normal oligomeric state and rescue enzyme function (Underhaug *et al*. 2012; Hole *et al*. 2015, 2016; Waløen *et al*. 2017).

## Acknowledgments and conflict of interest disclosure

Guri E. Matre, Ali J. Sepulveda, Kai Waløen and Sidsel E. Riise are thanked for their help and technical support. The authors are grateful for beam time and excellent beamline support at SWING beamline of SOLEIL Synchrotron. Alexander Shkumatov is acknowledged for his technical assistance during EM data collection and processing. This study was supported by Helse Vest (grant to Jan Haavik); Stiftelsen Kristian Gerhard Jebsen (grant to Jan Haavik), Seventh Framework Programme (grant No. 602805 to Jan Haavik). RL and GM acknowledge the Fonds voor Wetenschappelijk Onderzoek Vlaanderen (grant G0C1213N) and VUB Onderzoeksraad Strategic Research Project (grant SRP‐13) for research support. The authors have no conflict of interest to declare.

## Supporting information


**Figure S1.** Two‐step purification of recombinant hTH1 variants.
**Figure S2.** Protein yields and purification steps.
**Figure S3.** Gel filtration experiments showing the presence of octameric TH.
**Figure S4.** SDS‐PAGE of WT hTH1 (O & T) in the presence and absence of reducing agent.
**Figure S5.** Michaelis–Menten and substrate inhibition curves.
**Figure S6.** Suggested model for a filamentous WT hTH1.
**Table S1.** Tryptic peptide mapping followed by mass spectrometry.
