# Supplementary material for: The quaternary structure of human tyrosine hydroxylase: effects of dystonia‐associated missense variants on oligomeric state and enzyme activity
Source: J Neurochem. 2018 Dec 9;148(2):291–306. doi: 10.1111/jnc.14624 (PMC6587854; doi:10.1111/jnc.14624)
Supplement: Supplementary file 1 — Figure S1. Two‐step purification of recombinant hTH1 variants. Figure S2. Protein yields and purification steps. Figure S3. Gel filtration experiments showing the presence of octameric TH. Figure S4. SDS‐PAGE of WT hTH1 (O & T) in the presence and absence of reducing agent. Figure S5. Michaelis–Menten and substrate inhibition curves. Figure S6. Suggested model for a filamentous WT hTH1. Table S1. Tryptic peptide mapping followed by mass spectrometry. [file JNC-148-291-s001.pdf]

Supplementary material for

The quaternary structure of human tyrosine hydroxylase: effects of dystonia-associated missense variants on oligomeric state and enzyme activity

Peter D. Szigetvari[1,2], Gopinath Muruganandam[3,4], Juha P. Kallio[1], Erik I. Hallin[1], Agnete Fossbakk[2], Remy Loris[3,4], Inari Kursula[1,5,6], Lisbeth B. Møller[8], Per M. Knappskog[2,7,9], Petri Kursula[1,5] and Jan Haavik[1,2,*]

[1]Department of Biomedicine, University of Bergen, Bergen, Norway

[2]K.G. Jebsen Centre for Research on Neuropsychiatric Disorders, Department of Biomedicine, University of Bergen, Bergen, Norway

[3]Center for Structural Biology - Vlaams Instituut voor Biotechnologie (VIB), Brussels, Belgium

[4]Structural Biology Brussels, Department of Bioengineering Sciences, Vrije Universiteit Brussel (VUB), Brussels, Belgium

[5]Faculty of Biochemistry and Molecular Medicine, University of Oulu, Oulu, Finland

[6]Biocenter Oulu, University of Oulu, Oulu, Finland

[7]Department of Clinical Science, UiB, Bergen, Norway

[8]Applied Human Molecular Genetics, Kennedy Center, Department of Clinical Genetics, Copenhagen University Hospital, Rigshospitalet, Glostrup, Denmark

[9]Center for Medical Genetics and Molecular Medicine, Haukeland University Hospital, Bergen, Norway

*Author for correspondence:

Professor Jan Haavik

Department of Biomedicine

University of Bergen

Jonas Lies vei 91

5020 Bergen, Post box 7804

Norway

Phone: +47 55 58 64 32

Email: Jan.Haavik@uib.no

Supplementary figures

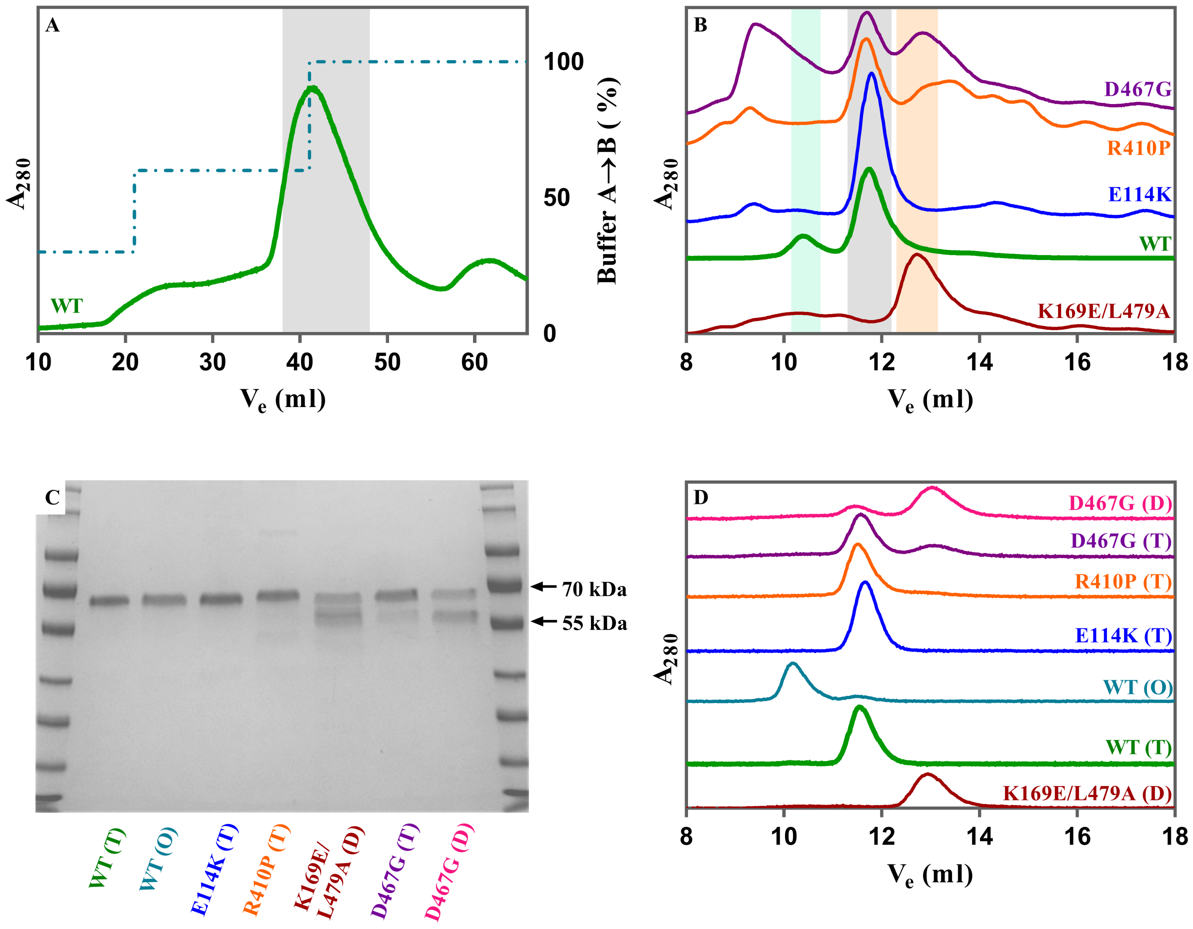

Supplementary Fig. 1: Two-step purification of recombinant hTH1 variants. This figure depicts the two stages of purification and evaluation of the untagged enzyme. **(A)** Heparin-Sepharose affinity purification using a step-gradient of increasing salt concentration from 150 mM (buffer A) to 500 mM (buffer B). Elution curve for the WT hTH1 is shown with collected volume (grey area). **(B)** Polishing gel filtration step. Mutant D467G displayed the highest propensity for aggregation. Collected volumes corresponding to different oligomerization states are shown as; cyan for octameric, grey for tetrameric and bright orange for dimeric species. **(C)** Quality assessment of hTH1 purification products by SDS-PAGE and analytical SEC **(D)** indicates high purity and a propensity for diverting from the typical quaternary structure as displayed by mutants but unexpectedly, also the WT enzyme.

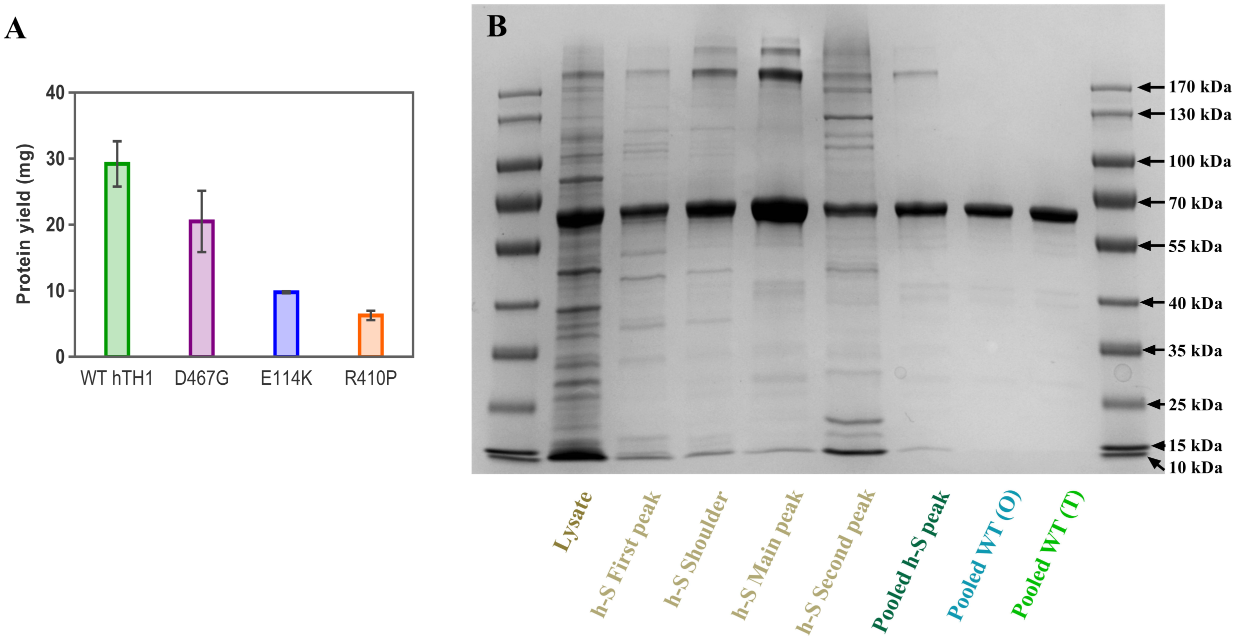

Supplementary Fig. 2: Protein yields and purification steps. (A) On average, 29.2 mg was yielded for the wild-type hTH1, 20.5 mg for D467G, 9.8 and 6.3 mg for E114K and R410P, respectively. Results are average of three separate purification efforts per hTH1 variant. **(B)** Examination of protein purity using SDS-PAGE with Coomassie staining (InstantBlue^{TM} from Expedeon). This figure depicts distinct stages during the purification process of the wild-type enzyme, including four fractions in lanes 3-6 from the heparin-Sepharose (h-S) affinity purification step, sampled at V_e = ~24, ~34, ~42 and ~62 ml, respectively (Supp. Fig. 1/A). Lane 7 contained the concentrated peak fractions from the affinity separation step (Supp. Fig. 1/A, gray area). Lanes 8 & 9 show the concentrated peaks of octameric and tetrameric hTH1 from the final gel filtration step (Supp. Fig. 1/B, cyan & gray areas).

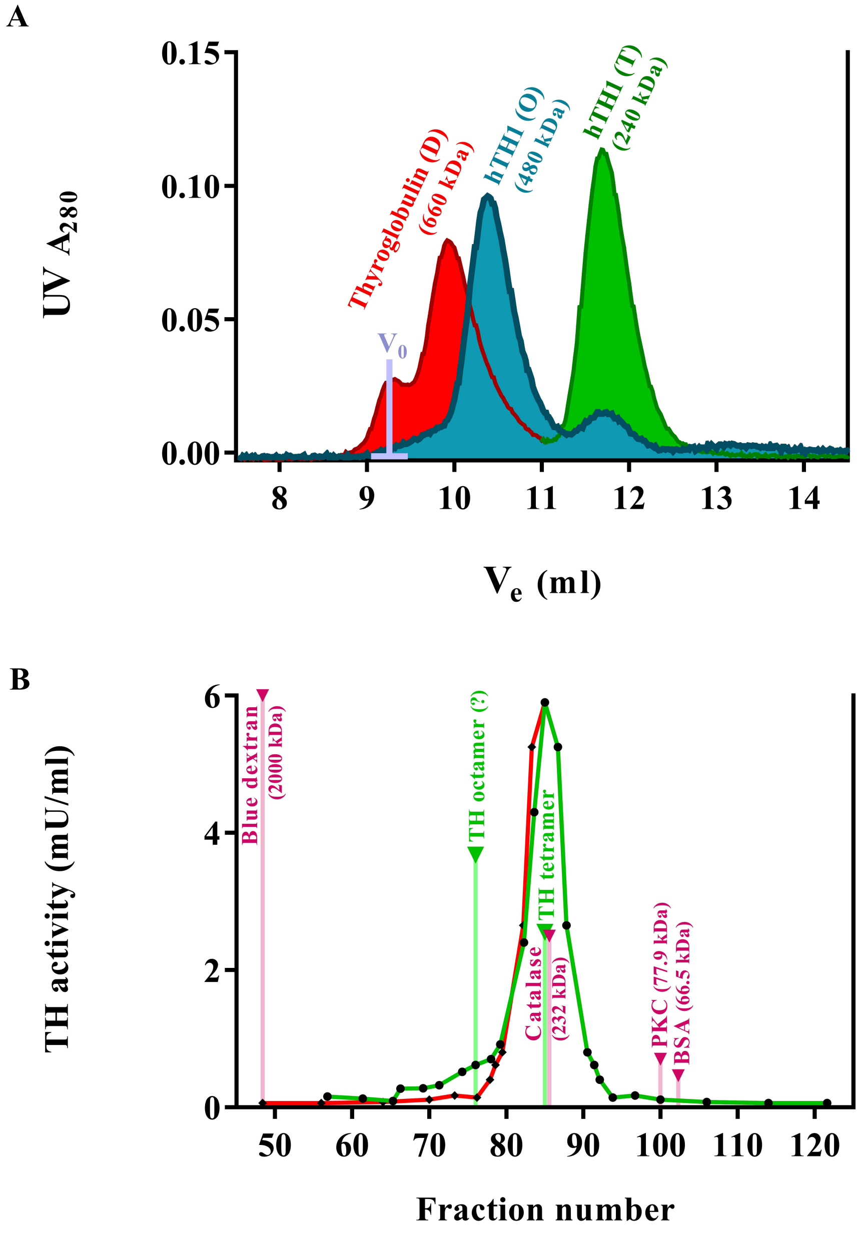

Supplementary Fig. 3: Gel filtration experiments showing the presence of octameric TH. (A) This experiment proved that the octameric peak is resolved by the Superdex 200 Increase SEC column and eluted after the void volume and the thyroglobulin standard. The void volume corresponds to ~ 9.2 ml. **(B)** Freshly processed tissue lysates from bovine adrenal medulla were subjected to centrifugation at 100 000 x g for 1 h at 4°C. Subsequently, the supernatant was subject to high-resolution SEC at 0.4 ml/min using TSK-G 3000 SW and TSK-4000 SW columns coupled in series (Toyo Soda, Japan, both columns 7.5 x 600 mm), while fractions were collected. The enzymatic activity profile that originated from these fractions showed a distinct 'shoulder' preceding the main peak (~ 15 % of total activity), indicating that high-order forms of TH are also present in freshly prepared lysates of mammalian tissue. The red curve shows the expected profile for a symmetrical protein peak. The elution volumes for various standards were determined using UV absorbance at 280 nm (magenta).

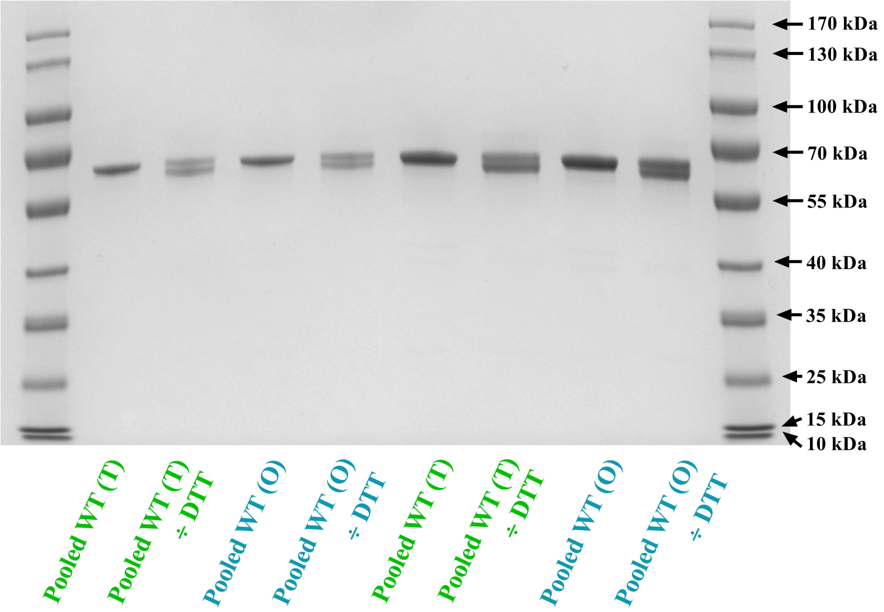

Supplementary Fig. 4: SDS-PAGE of WT hTH1 (O & T) in the presence and absence of reducing agent. Lane 1 and 10 accommodated pre-stained protein ladders. Lanes 2-5 and 6-9 contained ~ 0.5 and ~ 1.0 µg pure, gel filtrated hTH1. This experiment showed that secondary bands appeared under non-reducing conditions in cases of both the tetrameric and octameric forms, indicating that the purified enzyme is partially reduced.

hTH1	Tertiary assembly	MW bands (kDa)	First N-terminal peptides	Last C-terminal peptides
WT	T<br>O	~65<br>~65	17 – 24<br>13 – 37	477 – 497<br>477 – 497
E114K	T	~65	17 – 24	477 – 497
R410P	T>D	~67<br>~52	17 – 24<br>48 – 76	477 – 497<br>387 – 410
D467G	T>D<br>D>T	~65<br>~57	17 – 24<br>17 – 24	477 – 497<br>459 – 476
K169E/ L479A	D	<65	17 – 24	459 – 476

Supplementary Table. 1: Tryptic peptide mapping followed by mass spectrometry. Protein bands are shown in Supp. Fig. 1/C. Partial degradation was found in the lower MW bands of R410P and D467G as well as the normal MW band of K169E/L467K double mutant. However, as the SDS-PAGE in Fig. 3/B, D, F, G showed, only a proportion of dimeric mutant hTH1 assemblies is degraded.

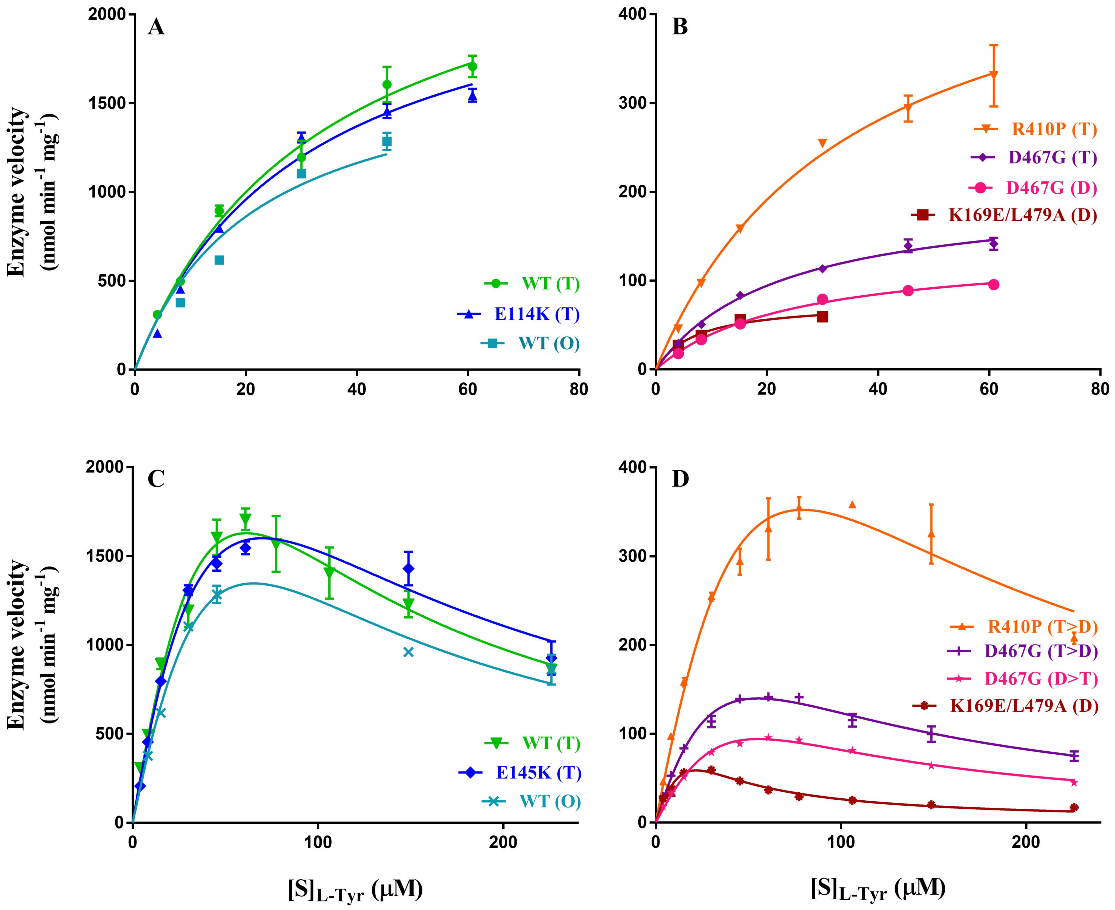

Supplementary Fig. 5: Michaelis-Menten and substrate inhibition curves. (A, B) Activity curves using the standard M-M equation with data points belonging to 0-60 μM L-Tyr concentrations. **(C, D)** Substrate inhibition shown on all variants.

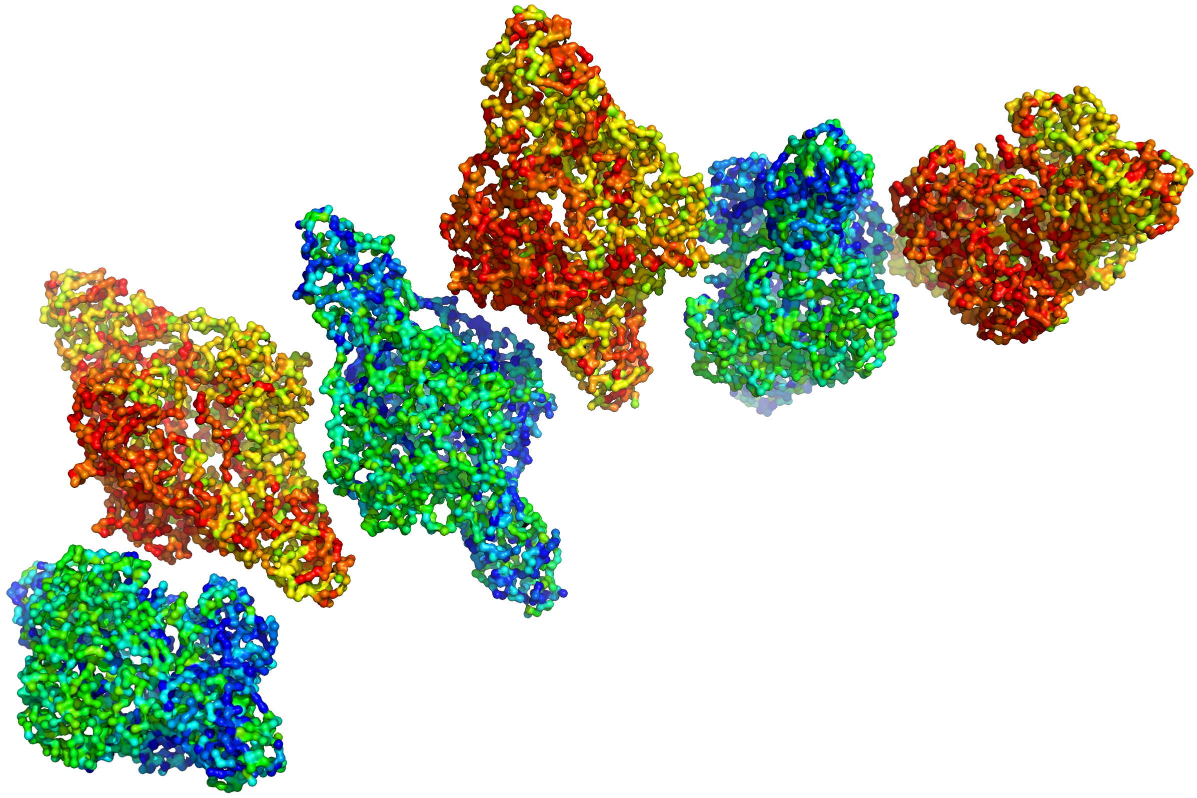

Supplementary Fig. 6: Suggested model for a filamentous WT hTH1. Based on the model for the WT octameric hTH1 assembly deduced from the SAXS data. The cartoon shows the superposition of several octamers into a chain-like structure, consistent with the TEM data.
